# Competencies of undergraduate physiotherapy education: A scoping review

**DOI:** 10.4102/sajp.v80i1.1879

**Published:** 2024-01-19

**Authors:** Tonderai W. Shumba, Ara Tekian

**Affiliations:** 1 Department of Occupational Therapy and Physiotherapy, Faculty of Health Sciences and Veterinary Medicine, University of Namibia, Windhoek, Namibia; 2 Department of Medical Education, Chicago College of Medicine, University of Illinois, Chicago, United States

**Keywords:** competencies, undergraduate physiotherapy, review, assessment strategies, milestones evaluation, Namibia

## Abstract

**Background:**

In recent years, the need for competency-based medical education has been emphasised. Each country needs a defined set of physiotherapy competencies from the associations and governing bodies.

**Objectives:**

Our review aimed to map competencies of undergraduate physiotherapy education and propose a context-specific competency framework for Namibia.

**Method:**

This scoping review was conducted following the Joanna Briggs Institute framework and was reported using the Preferred Reporting for Systematic Reviews and Meta-analysis Extension for Scoping Reviews. Qualitative direct content analysis utilising the five main competency domains from the WHO Rehabilitation Competency Framework was adapted.

**Results:**

Five main competency domains were proposed: practice, professional growth and involvement, learning and development, management and leadership, and research. Nineteen potential competencies were identified, and each competency has a set of knowledge and skills activities that is expected of each student.

**Conclusion:**

The proposed competencies still need to undergo expert consensus and content validation before they can be adopted and implemented in Namibia. Future studies can explore the perspectives and experiences of the faculty, students and clinicians on the current status of competency-based education of undergraduate physiotherapy programme in Namibia. Similarly, future studies can focus on possible assessment strategies that can be used for each competency and an evaluation framework for assessing milestones in student competencies from entry into clinical education to graduation.

**Clinical implications:**

The review proposed a context-specific competency framework for Namibia with a set of knowledge and skills activities that is expected of each student. The faculty can adopt these competencies and improve on their competency-based physiotherapy education.

## Introduction

### Rationale

In recent years, the need for competency-based medical education (CBME) has been emphasised. This shift to CBME has shortcomings of existing assessment (Harris et al. 2010), including strategies to assess the clinical competencies in the ever-changing medical education, characterised by the multidisciplinary approach among healthcare professionals, requiring patient safety, transparency and accountability (Lockyer et al. 2017).

Two key rationales for assessment are assessment of learning and assessment for learning. Competency-based medical education has shifted the rationale for assessment from assessment of learning to assessment for learning (Lockyer et al. 2017). In assessment for learning, active engagement of the learner forms the central role. Assessment should be performed *by* and *with* the learner (Sargeant et al. 2010). On the other side, assessment of learning focuses on the acquisition of knowledge or the demonstration of certain competencies in controlled settings. Given that these two rationales for assessments are different and the recent shift to CBME, our thinking should be on developing assessment rubrics that consider the effect of trainees’ competence on the quality of patient treatment outcomes (Kogan & Holmboe 2013).

There are many inconsistencies regarding the definition of competency (Kurunsaari et al. 2018). In 2010, following a systematic review by a group of educators (Frank et al. 2010), a more comprehensive definition of competence was provided:

The array of abilities across multiple domains or aspects of physician performance in a certain context. Statements about competence require descriptive qualifiers to define the relevant abilities, context, and stage of training. Competence is multi-dimensional and dynamic. It changes with time, experience, and setting. (p. 641)

Further, in 2020, a group of rehabilitation experts proposed a more recent definition: ‘Competencies are the observable abilities of a person, integrating knowledge and skills, as well as core values and belief in their performance of tasks’ (World Health Organization 2020:4).

Competency-based medical education is outcomes-based and is designed using a framework of competencies that drive its implementation, assessment and evaluation (Frank et al. 2010). To this end, it is important to identify the competencies expected in an undergraduate student during the development of the curriculum and assessment rubrics.

The University of Namibia has an undergraduate physiotherapy programme in its infancy, with its first cohort of graduates produced in May 2022. Further, the University of Namibia is the only institution in Namibia training physiotherapists. The undergraduate programme was developed through benchmarking against South African universities. There are many contextual factors influencing the application of competency-based physiotherapy education. Some factors that hinder the application of competency-based education include the number and type of accredited clinical settings for student rotations, the number of speciality clinicians in hospitals to assist with clinical supervision, and the number and frequency of clinical assessments.

The current physiotherapy education system has the consequence of producing a graduate who is likely to lack clear-cut competencies that are responsive to the Namibia and international rehabilitation standards. This may run the risk of having a clinician who is not adequately ready for delivering quality physiotherapy services responsive to the Namibian population, thus compromising patient safety and treatment outcomes. Further, there is a consequence of breeding a lack of common assessment language among Namibia’s students, faculty, clinicians, external examiners and licensing bodies. To this end, this practice can lead to a lack of cohesion among professionals, thus hampering teamwork in patient care.

Globally, various physiotherapy associations and governing bodies have set contextual competencies expected from each physiotherapy graduate. In Namibia, most of the regulations and guidelines were developed prior to this physiotherapy programme and have not yet been reviewed to include contextual competencies required for physiotherapy education. Having practised physiotherapy in Namibia for over 15 years in Namibia, the authors believe that the lack of a defined set of competencies from the associations and governing bodies has led to trial and error practice when it comes to structuring clinical blocks and finding the best-fit formula for assessments.

### Objectives

The main aim of our review was to map the undergraduate physiotherapy education competencies and propose a context-specific competency framework for Namibia. There is no evidence of any scoping or systematic literature review on undergraduate physiotherapy competencies in Africa. Thus, our review aims to answer the main research question: what evidence is available on undergraduate physiotherapy education competencies? Further, the review intends to answer the secondary question: what are the contextually relevant competencies for physiotherapy education in Namibia? However, the competencies will require to undergo expert consensus and content validation before physiotherapy educators can use them to review the learning outcomes for their curriculum (World Physiotherapy 2021). Moreover, the Allied Health Professions Council of Namibia can adapt them as an evaluation of physiotherapists for registration.

## Research methods and design

This scoping review followed the Joanna Briggs Institute (2015) methodology for scoping reviews. The Preferred Reporting for Systematic Reviews and Meta-analysis Extension for Scoping Reviews (PRISMA-ScR) (Tricco et al. 2018) was followed for reporting the findings.

### Protocol and registration

The review protocol was registered with the Open Science framework and can be accessed at the following link: https://osf.io/4r6tm/.

### Eligibility criteria

#### Type of participants

All studies reporting on the general competencies and physiotherapy domain-specific competencies for undergraduate and entry-level physiotherapy programmes will be included.

#### Concept

The concept of this study is undergraduate physiotherapy competencies. For this study, ‘Competencies are the observable abilities of a person, integrating knowledge and skills, as well as core values and belief in their performance of tasks’ (World Health Organization 2020:4). This study adopted the structure and adapted the contents of the WHO Rehabilitation Competency Framework (RCF). This WHO RCF ‘is a model that communicates the expected or aspired performance of the rehabilitation workforce across professions, specialisations and settings to enable quality care and service delivery’ (World Health Organization 2020:1). This framework was chosen as it resonates with the vision of the Rehabilitation 2030 Initiative.

#### Context

All studies reporting competencies for undergraduate physiotherapy at the global level in a rehabilitation centre, healthcare setting and community setting were considered. Understanding the competencies required of a physiotherapy student in any setting is important.

#### Types of sources

All types of primary studies, all types of reviews, grey literature (websites, guidelines, theses) and books from electronic databases were included in our review.

#### Time frame

The time frame chosen was from January 1990 to May 2023.

### Exclusion criteria

The exclusion criteria include the following:

- All articles with titles and abstracts not in English. This is because English was used in the first step for screening titles.

### Information sources

The following electronic databases were searched: Cochrane Central Register of Controlled Trials, Cochrane Database of Systematic Reviews, PubMed Central, EBSCOHost, Science Direct, Wiley Online Library and Scopus. Further data were sourced from websites of associations and governing bodies globally including World Physiotherapy, World Health Organization, European Physiotherapy, Canada, America, Spain, the United Kingdom, Australia, New Zealand, South Africa and Namibia.

### Search

An initial limited search of two online databases including the Cochrane Library and PubMed Central was conducted to guide the main search strategy. The search strategy utilised Medical Subject Headings (MeSH) terms and keywords related to ‘undergraduate physical therapy’ OR ‘undergraduate physiotherapy’ AND ‘competent*’ (Appendix 1).

The first reviewer (T.S.) did a comprehensive search in electronic databases, including Cochrane Central Register of Controlled Trials, Cochrane Database of Systematic Reviews, PubMed Central, EBSCOHost, Science Direct, Wiley Online library and Scopus with publication coverage from January 1990 to May 2023. The second reviewer (A.T.) used the exact keywords and Boolean operators used by the first reviewer to retrieve the same number and type of studies from the included databases. Additionally, the reference lists of retrieved articles were manually searched for possible relevant studies.

### Selection of sources of evidence

The Joanna Briggs Institute methodology (The Joanna Briggs Institute 2015) guided the search selection of sources. The selection was based on title, abstract and full-text screening. The first author (T.S.) screened the titles of the retrieved studies. Further, T.S. and A.T. independently screened the abstracts against the inclusion criteria. The studies retained for full text were then independently assessed for eligibility by two reviewers (T.S. and A.T.). The reference lists from all the full-text studies were searched for potential additional study. Full-text studies not meeting the inclusion criteria were excluded and reasons were provided (Appendix 2). Disagreements in the selection process were resolved through discussions.

### Data charting process

Two reviewers extracted data using an adapted data extraction tool from the Joanna Briggs Institute (The Joanna Briggs Institute 2015). The following headings were used on the data extraction sheet: author(s), year of publication, place of publication, process followed in identifying competencies, type of competencies and list of competencies. The first reviewer (T.S.) initiated the data extraction and the second reviewer (A.T.) conducted independent verification. Discussions were held to resolve any disputes.

### Data items

The following comprise a list and definitions of all variables for which data were sought and any assumptions and simplifications made.

- *Author(s)*: It indicates the author(s) who published the document. A document may mean a peer-reviewed article, a framework, a guideline and a report.
- *Year of publication:* It indicates the year the document was published.
- *Place of publication:* This is the place where the document was published. This was sought by country and later by region.
- *Process followed in identifying competencies:* This identifies the methodology followed, the stakeholders or participants involved and the completion time frame. We assumed that the process indicated the results’ validity and reliability.
- *Type of competencies:* This defined the competencies including general competencies for physiotherapists, competencies for physiotherapy registration and licensing, physiotherapy exit competencies, and domain-specific competencies (e.g. pain management).
- *List of competencies:* This is a pool of competencies that were identified across the documents.

### Synthesis of results

Firstly, descriptive statistical analysis was conducted on the number of studies, trend of studies, geographical locations, process followed in identifying competencies and type of competencies. Secondly, conventional content analysis (Namey et al. 2008) was employed to analyse the competencies. This was conducted by reviewing the documents and highlighting text describing competence. These data were extracted verbatim and added to an Excel sheet for coding. The final subthemes represented the competencies and the codes indicated activities.

Finally, direct content analysis utilising the WHO RCF (World Health Organization 2020) was adopted for its structure and adapted for its content. Thus, the domains of the WHO RCF were adopted as the main themes which included practice, professionalism, learning and development, management and leadership, and research. The content was determined by the results from convectional content analysis where the subthemes represented the competencies and the codes indicated skills and knowledge-specific activities of that competence.

### Ethical considerations

The Ethical Clearance Certificate was issued by the University of Namibia Decentralized Ethics Committee (DEC) in accordance with the University of Namibia’s Research Ethics Policy and Guidelines (SAH08/22 – 24/07/2022).

## Results

### Selection of sources of evidence

**FIGURE 1:**
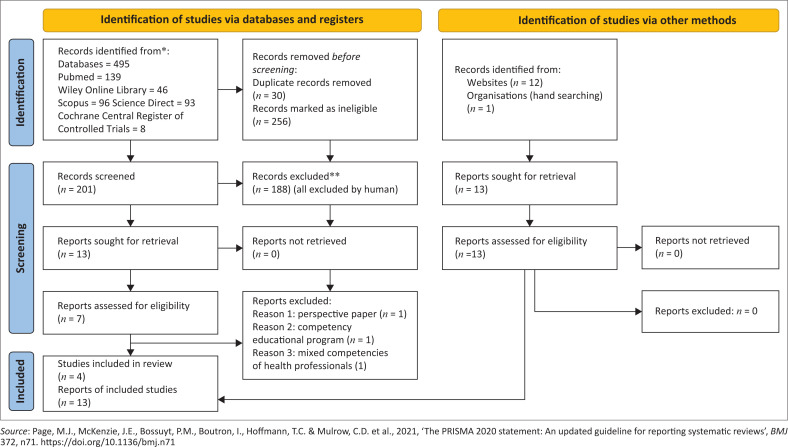
PRISMA flow diagram.

The initial search from electronic databases identified 495 publications. A total of 30 duplicates were removed and 256 were found to be ineligible before screening. After the abstract screening of 201 articles, 188 were excluded and 13 were retained for full text screening. A total of four articles were finally included. A search of websites and hand searching in organisations retrieved a total of 13 documents (frameworks [10], scope of practice [1], regulation for licensing [1], curriculum [1]). The study finally included 16 documents for analysis. This is depicted in the Preferred Reporting Items for Systematic Reviews and Meta-Analyses (PRISMA) flow diagram (Peters et al. 2015) (Figure 1).

### Characteristics of individual sources of evidence

The types of publication were competence frameworks (10), peer-reviewed articles (4), scope of practice (1), regulations for licensing (1) and curriculum (1). Summary of the documents extracted is shown in Table 1.

**TABLE 1:** Summary of documents selected.

Authors	Title	Year of publication	Source	Type of publication	Region and/or country
World Health Organization	Rehabilitation Competency Framework	2020	WHO Website (https://apps.who.int/iris/handle/10665/338782)	Framework	Geneva
World Physiotherapy	Physiotherapist Education Framework	2021	World Physiotherapy Website (https://world.physio/news/world-physiotherapy-publishes-framework-physiotherapist-education)	Framework	Global
Australian Physiotherapy Association & Australian College of Physiotherapists (2023)	Physiotherapy Competency Framework	2023	Australian Physiotherapy Association	Framework	Australia
Physiotherapy Board of Australia & Physiotherapy Board of New Zealand	Physiotherapy Practice Thresholds in Australia and Aotearoa New Zealand	2015	Physiotherapy Board of Australia (https://physiocouncil.com.au/wp-content/uploads/2017/10/Physiotherapy-Board-Physiotherapy-practice-thresholds-in-Australia-and-Aotearoa-New-Zealand.pdf)	Framework	Australia/New Zealand
Chartered Society of Physiotherapy	Physiotherapy Framework: putting physiotherapy behaviours, values, knowledge and skills into practice	Updated 2020	Chartered Society of Physiotherapy website (Free CPD resources for CSP members | The Chartered Society of Physiotherapy)	Framework	United Kingdom
Physiotherapy Board of New Zealand	Physiotherapy Standards Framework	2018	Physiotherapy Board of New Zealand (https://www.physioboard.org.nz/)	Framework	New Zealand
European Region of the WCPT	Expected Minimum Competencies for an Entry-Level Physiotherapist in the European Region	2018	European Region of WCPT website (https://www.erwcpt.eu/file/251)	Framework	European Region
National Physiotherapy Advisory Group	Competency Profile for Physiotherapists in Canada	2017	Physiotherapy Advisory Group website (https://peac-aepc.ca/pdfs/Resources/Competency%20Profiles/Competency%20Profile%20for%20PTs%202017%20EN.pdf)	Framework	Canada
American Physical Therapy Association	Core Competencies of a Physical Therapist Resident	2020	American Physical Therapy Association Website (https://www.apta.org/for-educators/core-competencies-pt-resident)	Framework	United States
Unam Physiotherapy Programme	Transformed Curriculum: Graduate Competencies and Employability	2022	Hard copy (Department of Occupational Therapy & Physiotherapy)	Curriculum competencies	Namibia
*Allied Health Professions Act, 2004*	Regulations Relating to the Minimum Requirements of Study for Registration as a Physiotherapist	2004	AHPCN website (https://www.hpcna.com/images/councils/allied/Minimum%20requirements%20and%20registration/4581%20Physio%20minimum%20requirement.pdf)	Regulations for registration	Namibia
*Allied Health Professions Act, 2004*	Scope of Practice for Physiotherapists	2004	AHPCN website (https://www.hpcna.com/images/councils/allied/Scope%20of%20Practice/4502%20Regulations%20relating%20to%20Scope%20of%20Physio’s.pdf)	Scope of practice	Namibia
Professional Board for Physiotherapy, Podiatry and Biokinetics in South Africa	Minimum Standards for Training: Physiotherapy	Updated February 2022	HPCSA website (https://www.hpcsa.co.za/?contentId=0&menuSubId=51&actionName=For%20Professionals)	Framework	South Africa
Díaz-Mohedo et al.	Rubric for the Evaluation of Competencies in Traumatology in the Degree of Physiotherapy: Delphi Approach	2021	*BMC Medical Education* Journal	Article	Spain
Martin R, Mandrusiak A, Lu A, Forbes R	Competencies for Entry-Level Rural and Remote Physiotherapy Practice: A Delphi Approach	2021	*Rural and Remote Health*-Journal	Article	Australia
Forbes et al.	Identification of Competencies for Patient Education in Physiotherapy Using a Delphi Approach	2018	*Physiotherapy Journal* (https://www.physiotherapyjournal.com/action/showPdf?pii=S0031-9406%2817%2930053-6)	Article	Australia
Hoeger et al.	An Interprofessional Consensus of Core Competencies for Prelicensure Education in Pain Management: Curriculum Application for Physical Therapy	2014	Physical Therapy Journal	Article	America

WCPT, World Confederation of Physical Therapy.

### Results of individual sources of evidence

#### Year of publication

**FIGURE 2:**
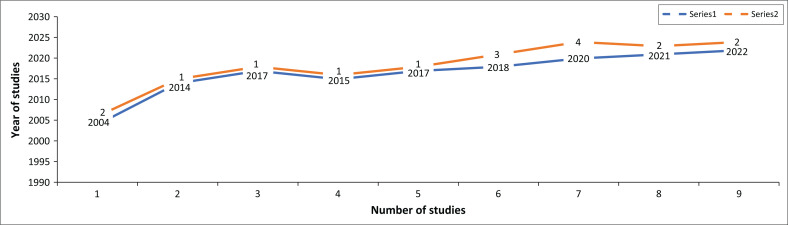
Year of publication.

Figure 2 indicates there has been a growing interest in competency-based education and practice with a peak in the year 2020.

#### Geographical location

Most of the documents retrieved were from Oceania (5). None were retrieved from Asia and South America (Table 2).

**TABLE 2:** Geographical location.

Region	Number
Global	2
Oceania (Australia and/or New Zealand)	5
European Region Group (all European countries plus the United Kingdom)	3
North America (the United States and Canada)	2
Southern Africa (Namibia and South Africa)	4

**Total**	**16**

#### Process followed in identifying competencies

Most of the global competencies were developed using country consultations, technical working groups and delphi consensus studies (Table 3).

**TABLE 3:** Process followed in identifying competencies.

Process	Frequency of documents
Multi-country consultation	3
Regional country consultation (four countries)	1
Country consultation	9
Technical working groups	7
Delphi consensus studies	6
Policy reviews	3

### Synthesis of results

Our review aimed to map the competencies of undergraduate physiotherapy education and propose a context-specific competency framework for Namibia. Five domains were adopted from the WHO RCF (World Health Organization 2020). Nineteen potential competencies were identified from various frameworks and studies globally. Each competency has a set of activity-specific knowledge and skills expected of each student. A sample of how the competencies were identified and aligned to the domains of the WHO RCF is shown in Table 4 and the proposed context-specific competency framework for Namibia is shown in Appendix 3.

**TABLE 4:** Competence identification and alignment to the domains of WHO Rehabilitation Competency Framework.

Authors	Process followed in identifying World Physiotherapy competencies	Type of World Physiotherapy competencies	List of the World Physiotherapy competencies	WHO Rehabilitation Competency Domain they align
World Physiotherapy	Several documents from the electronic databases, World Physiotherapy, physiotherapy associations worldwide, and those from other organisations developed by the European Union have been consulted in the preparation of this document.	Minimum expected competencies for an entry-level physiotherapist in the European Region.	Assessment competencies Diagnostic competencies Intervention competencies Professional and interprofessional competencies Health promotion and prevention competencies Research and evidence-based competencies Education and learning competencies Management competencies	**Practice** (assessment competencies, diagnostic competencies, intervention competencies, health promotion and prevention competencies) **Professionalism** (professional and interprofessional competencies) **Learning and development** (education and learning competencies) **Management and leadership** (management competencies) **Research** (research and evidence-based competencies)

*Source:* American Physical Therapy Association 2020; Australian Physiotherapy Association &Australian College of Physiotherapists 2023; Chartered Society of Physiotherapy 2020; Díaz-Mohedo et al. 2021; Edgar et al. 2020; Forbes, Mandrusiak & Smith 2018; Health Professions Council of South Africa 2022; Hoeger et al. 2014; Martin, Mandrusiak & Lu 2021; National Physiotherapy Advisory Group (NPAG) 2017; Physiotherapy Board of Australia & Physiotherapy Board of New Zealand 2015; Republic of Namibia 2002, 2018; University of Namibia 2022; Ursula et al. 2017; WCPT European Region 2018; Physiotherapy Board of New Zealand 2018; World Confederation for Physical Therapy 2011; World Health Organization 2020; World Physiotherapy 2021

## Discussion

Competency frameworks have the potential of providing a shared language, unifying and harmonising rehabilitation professionals (World Health Organization 2020). Our review aimed to map the competencies of undergraduate physiotherapy education and propose a context-specific competency framework for Namibia. The study synthesised several global entry-level physiotherapy competencies enshrined in competency frameworks, scope of practice, regulation for licensing and curriculum. The WHO RCF (World Health Organization 2020) underpinned the data analysis and the subsequent development of the proposed context-specific competency framework for Namibia (Appendix 3). Thus, five domains of the WHO RCF were adopted as the main themes which included practice, professionalism, learning and development, management and leadership, and research. Nineteen competencies were identified from convectional content analysis of the final included documents, and they represented the subthemes. The codes indicated skills-specific activities and knowledge-specific activities of that competence. Notably, the proposed competencies are intended to supplement the current competencies and to add clarity, strength and content.

It is critical to track the student’s progress from entry to graduation based primarily on competency acquisition, where time becomes a resource for education and not a determinant for graduation. There is a need for guidance on a student’s observable behaviours and other attributes at a significant point in development to be a physiotherapist (Edgar et al. 2020). Notably, adopting a competency-based undergraduate physiotherapy education has the potential of de-emphasising a time-based credentialing system where graduation to physiotherapist would be hinged on the acquisition of skills rather than time frames (Frank et al. 2010).

The adopted five domains allowed for the thematic grouping of the synthesised competencies and their accompanying knowledge and skill statements. The most prominent domain of practice entails competencies hinged on the interaction between the undergraduate physiotherapy student and the patient and family (World Health Organization 2020). The physiotherapy practitioner role is critical to the functioning of the physiotherapist (Australian Physiotherapy Association & Australian College of Physiotherapists 2023). Seven context-specific competencies were identified for this domain, including assessment, diagnosis, intervention, communication, clinical reasoning, health promotion and prevention. While most of these competencies converge in many settings, one key competence relevant for the resource-constrained setting of Namibia is health promotion and prevention driven by the WHO community-based rehabilitation strategy (WHO et al. 2010). Thus, physiotherapists should serve as health advocates (Australian Physiotherapy Association & Australian College of Physiotherapists 2023), facilitate behaviour change (European Region Physiotherapy 2018) and transfer knowledge and skills on rehabilitation to people with disabilities, their families and the community in remote areas where access to health services is limited (WHO 2010). Community-based education becomes a priority for countries seeking quality primary healthcare, where their students are transformed into sensitive and responsive health professionals with a lens of social determinants of health (Claramita et al. 2019).

During the training period, the students must understand and practise professionalism and involvement. The review identified competencies related to ethics, collaboration and quality improvement. The undergraduate preclinical programme should prepare students for their clinical placement regarding ethical conduct and communication (Wijbenga, Bovend’Eerdt & Driessen 2018). Students must collaborate within their professions and other disciplines to ensure quality treatment outcomes. The ability to appreciate the roles of other disciplines allows students to collectively establish common client-centred goals. It thus can manage conflict that may arise (Australian Physiotherapy Association & Australian College of Physiotherapists 2023).

Self-learning and development is one critical domain. Thus, the students need to demonstrate competencies of self-learning as well as promoting the learning of others. Students are required to develop their professional development plan and be able to reflect on practice and seek guidance as needed (World Health Organization 2020). Self-directed learning promotes student autonomy and responsibility for learning in an educational climate that reduces anxiety (Akulwar-Tajane & Varghese 2021). Encouraging students to adopt self-directed learning can potentially benefit them by embracing the new blended learning approaches (Akulwar-Tajane & Varghese 2021).

Importantly, leadership and management can enable the students to acquire competencies in managing a rehabilitation team, service delivery, monitoring and evaluation. In Namibia, physiotherapy graduates must be deployed in remote areas where they need to demonstrate these competencies. To this end, physiotherapy education is required to equip the students with the competencies that will enable them to independently manage a rehabilitation department upon graduation (World Physiotherapy 2021).

Central to any profession is evidence-based practice which is driven by research. Physiotherapists are required to apply clinical reasoning underpinned by evidence-based practice (Wijbenga et al. 2018). Understanding the research process is critical at the undergraduate level. To advance the profession through evidence generation, dissemination and integration, entry-level physiotherapists are required to have a fair understanding of designing basic operational research and to be able to disseminate through various platforms (World Health Organization 2020).

Competency-based education emphasises a learner-centred approach, where learners take charge of their learning, and de-emphasises a time-based approach (Frank et al. 2010). Most medical education programmes are time-bound and thus defeat the ideology of allowing students to achieve their competency at their own pace (Frank et al. 2010). However, students can acquire competencies at different times, and it is thus important for the education system to be flexible to adjust the time spent on some tasks to accommodate these learners (Carraccio et al. 2008). For example, the competency of clinical reasoning should be approached with caution as it also follows the normal developmental pattern of a baby from learning to crawl and then progressing to standing and walking. The academic supervisors and clinicians must understand that their role involves nurturing the student’s growth by facilitating the process using new teaching methods and assessment techniques to be utilitarian and efficient.

Our review is a first step towards a competency framework for physiotherapy education in Namibia. It is expected that the physiotherapy educators, clinicians, examiners, students and registration bodies can reflect on their practice and identify areas that need improvement regarding competency-based education. The proposed competency framework will require expert consensus and content validation before adoption.

## Limitations

Firstly, the reviewer only has access to databases hosted by the University of Namibia. Secondly, articles that had abstracts not in English were not included in the review. This could have potentially missed some crucial studies in other languages.

## Conclusion

To advance physiotherapy education in Namibia, our review synthesised various global physiotherapy competency frameworks, scope of practice, regulation for licensing and original studies to propose a contextually specific competency framework for Namibia. The proposed competencies still need to undergo expert consensus and content validation before they can be adopted and implemented in Namibia. Significantly, this contextualised framework can be utilised in several ways, including proposing to the Allied Health Professions Council of Namibia to guide the competencies required for physiotherapy registration; the University of Namibia physiotherapy programme can use it to convey their courses’ learning outcomes so that they are responsive to the needs of the population, and the Ministry of Health and Social Services can use it to plan human resource recruitments and evaluation.

## Appendix 1

**TABLE S1:** Search strategies for the electronic databases, date of the last search: 20 May 2023.

Key terms			
Undergraduate physiotherapy		‘undergraduate physical therapy’ OR ‘undergraduate physiotherapy’	
Competence		competenc*	
# Database		Query	Results
1	PubMed (*Time filter* open)	((‘undergraduate physical therapy’) OR ‘undergraduate physiotherapy’) AND competenc* Sort by: Best Match Filters: Publication date open	139
2	Wiley Online Library (*Time filter* open, Search all databases)	‘((“undergraduate physical therapy”) OR “undergraduate physiotherapy”) AND competenc*’	46
3	Scopus (*Time filter* open)	‘((“ undergraduate AND physical AND therapy”) OR “undergraduate AND physiotherapy ”) AND competenc*’	96
4	ScienceDirect (*Time filter* open)	‘undergraduate physical therapy’ OR ‘undergraduate physiotherapy’ AND competence	93
5	*Cochrane* Central Register of Controlled Trials (*Time filter* open)	‘undergraduate physical therapy’ OR ‘undergraduate physiotherapy’ AND competence	8
	EBSCOhost (*Time filter* open, Search all databases)		113

		**Total**	**495**

**Table:** Search on websites

Source	Type of publication
WHO Website (https://apps.who.int/iris/handle/10665/338782)	Framework
World Physiotherapy Website (https://world.physio/news/world-physiotherapy-publishes-framework-physiotherapist-education)	Framework
Physiotherapy Board of Australia (https://physiocouncil.com.au/wp-content/uploads/2017/10/Physiotherapy-Board-Physiotherapy-practice-thresholds-in-Australia-and-Aotearoa-New-Zealand.pdf)	Framework
Chartered Society of Physiotherapy website (Free CPD resources for CSP members | The Chartered Society of Physiotherapy)	Framework
Physiotherapy Board of New Zealand (https://www.physioboard.org.nz/)	Framework
European Region of WCPT website (https://www.erwcpt.eu/file/251)	Framework
Physiotherapy Advisory Group website (https://peac-aepc.ca/pdfs/Resources/Competency%20Profiles/Competency%20Profile%20for%20PTs%202017%20EN.pdf)	Framework
American Physical therapy Association Website (https://www.apta.org/for-educators/core-competencies-pt-resident)	Framework
AHPCN website (https://www.hpcna.com/images/councils/allied/Minimum%20requirements%20and%20registration/4581%20Physio%20minimum%20requirement.pdf)	Regulations for registration
AHPCN website (https://www.hpcna.com/images/councils/allied/Scope%20of%20Practice/4502%20Regulations%20relating%20to%20Scope%20of%20Physio’s.pdf)	Scope of practice
HPCSA website (https://www.hpcsa.co.za/?contentId=0&menuSubId=51&actionName=For%20Professionals)	Framework
**Total: 11**	
**Hand searching**	
Hard copy (Department of Occupational Therapy and Physiotherapy: University of Namibia)	Curriculum
**Total: 1**	

## Appendix 2

### List of excluded articles

1. Timmerberg, J.F., Chesbro, S.B., Jensen, G.M., Dole, R.L. & Jette, D.U., 2022, ‘Competency-based education and practice in physical therapy: It’s time to act!’, *Physical Therapy* 102(5), pzac018. https://doi.org/10.1093/ptj/pzac018.
  *Reason for exclusion: a perspective paper promoting competencies*.
2. Vissers, D., Daele, U.V., De Hertogh, W., De Meulenaere, A. & Denekens, J., 2014, ‘Introducing competency-vased education based on the roles that physiotherapists fulfil’, *Journal of Novel Physiotherapy and Physical Rehabilitation* 1(3), 110.
  *Reason for exclusion: firstly, for the development of a competency-based educational programme for physiotherapy and secondly to present the results of the evaluation of the competency-based programme*.
3. Verma, S., Paterson, M. & Medves, J., 2006, ‘Core competencies for health care professionals: What medicine, nursing, occupational therapy, and physiotherapy share’, *Journal of Allied Health* 35(2), 109–115.
  *Reason for exclusion: shared model not specific to physiotherapy*.

## Appendix 3

**APPENDIX 3:** List of domains, competencies and activity-specific knowledge and skills.

Domain	Key competencies	Activity-specific knowledge	Activity-specific skills
Practice	Assessment	Informed consent Definition and legal and ethical implications of written and verbal informed consent Approaches to determining a person’s decision-making capacity Policies and practices governing how, when and from whom informed consent is obtained and documented, including when a person does not have decision-making capacity	Informed consent Interviewing Explaining processes, risks, benefits and potential outcomes to people and their families with various levels of health literacy
		Conducting patient assessments Potential sources of information for gathering a person’s history Type and purpose of information to be collected and recorded Indications that a person is in need of protection measures and how these are initiated Methods of assessment, such as testing, measurement and evaluation, and when these are applied Assessment options relevant to scope of practice and considerations for selection Psychometric properties of assessment tools relevant to scope of practice Risks associated with conducting assessments relevant to scope of practice and how these are managed Identify client-specific precautions, contraindications and risks Resource requirements for assessments relevant to scope of practice Real and potential impact of health, personal and environmental factors on assessment results Knowledge of cultural humility and cultural competence Observe diversity in persons	Conducting assessments Accurately, comprehensively and efficiently performs a speciality-specific examination in simple and complex clinical situations Perform, in patients who require so, an evaluation (visual and/or instrumental) of the movement of the patient (range, quality, strength) and its possible alterations, recording such results adequately Assess and record pain, in patients who require so, through validated instruments, and identify the type of pain observed (nociceptive, visceral, neuropathic or chronic dysfunctional) When appropriate, conduct a neurological assessment of the patient through a superficial and deep sensitivity test and a reflex test, recording such information with proper terminology and interpreting the results adequately Setting up and using equipment and consumables relevant to scope of practice Positioning a person to conduct an assessment Prescribing and/or delivering different types of assessments relevant to scope of practice Adapting assessments to a person’s needs Scoring and interpreting assessment results Ensure patient comfort and dignity during assessment Understand the multidimensional nurture of pain – what is pain? Conduct pain assessment and measurement – how is pain recognised? Use of valid and reliable tools
		Documentation Policies and procedures for the collection, storage and access of information Type and purpose of information to be collected and documented Standardised formats for documenting information	Documentation Organising and filing information
	Diagnosis	Interpret diagnostic imaging and laboratory test results Interpret and evaluate assessment findings, and provide a physiotherapy diagnosis	Communicate findings of test in an appropriate language Accurately, comprehensively and efficiently performs a speciality-specific evaluation in simple and complex clinical situations to establish a diagnosis and prognosis Communicate the physiotherapy diagnosis to patient and family
	Intervention	Developing and adapting plans Methods of establishing priorities and desired outcomes of a person and their family Intervention options relevant to scope of practice and considerations for selection Frequency and duration typically required for interventions relevant to scope of practice to achieve desired outcomes Range of health interventions potentially involved in a person’s treatment, relevant to scope of practice, and their implications for a rehabilitation plan Typical care pathways relevant to scope of practice Methods of constructing a rehabilitation plan, including who should be involved Indications of the need to, and approaches of, adapting a rehabilitation plan	Developing and adapting plans Establishing client-centred goals and developing an individualised plan of evidence-based intervention using a context-specific, active, functional rehabilitation approach in full collaboration with the client/carers Setting and reviewing goal Constructing a rehabilitation plan
		Implementing plans Intervention options relevant to scope of practice and considerations for selection Evidence base for interventions relevant to scope of practice Risks associated with implementing interventions and how these are managed Indications and contraindications for the implementation of interventions relevant to scope of practice	Implementing plans Employ safe client handling techniques Apply assessment and intervention procedures in a manner that enhances the client’s safety and comfort Monitor and respond to client’s physical and emotional state throughout care Identify and respond to near misses and adverse events
		Potential modes of intervention, such as group sessions, mHealth and telerehabilitation, and considerations for selection Existing and emerging technologies for interventions relevant to scope of practice Resource requirements for interventions Methods and techniques for implementing interventions, including how to use relevant equipment and consumables Methods of adapting or grading interventions to a person Methods of training and supporting family members or caregivers to deliver or assist with interventions Timing for which interventions relevant to scope of practice should be conducted to achieve desired outcomes Frequency and duration of an intervention relevant to scope of practice to achieve desired outcomes Reasons for noncompliance with rehabilitation plans and methods of maximising compliance	Correctly prescribe and control physical exercise in its different modalities (isometric, concentric and eccentric isotonic, functional, motor control, conscious movement, etc.), adequately selecting among them to achieve the best result according to the objective set in a musculoskeletal dysfunction Prescribe, perform and/or control the proprioceptive and neuromuscular re-education techniques in their different phases, adequately selecting the techniques according to the evolutionary phase of the patient to achieve the best result based on the objective Correctly propose coherent physiotherapeutic objectives in the short, medium and long term, considering the pathology and the individuality of the user and his/her expectations and preferences Plan the treatment based on the objectives set, attending to the criteria of adequacy, validity and efficiency, considering risks and contraindications and efficiently managing the treatment time Correctly perform a bandaging (functional, neuromuscular, compressive), knowing the best choice among the different materials and techniques to achieve the best result according to the objective set Correctly carry out an application of electrotherapy (motor electrostimulation, TENS, galvanic current, magnetotherapy, laser, shock waves), selecting among the different parameters, and establishing dosimetries and application times, etc., to achieve the best result according to the objective set Correctly carry out the different techniques of manual therapy: massage therapy (decontraction, bowel evacuation, cicatrisation massage, Cyriax), adequately selecting among them and correctly executing them to achieve the best result according to the objective set Correctly carry out an application of thermotherapy (paraffin baths, MW, PSWT, radiofrequency, etc.), selecting among the different parameters, and establishing dosages and application times, etc., to achieve the best result according to the objective set Carry out the procedure of progressive loading and gait reeducation in patients who require so due to the lower limbs injuries Correctly carry out the following techniques of manual therapy: neuromuscular techniques, adequately selecting among them to achieve the best result according to the objective set Correctly carry out the following techniques of manual therapy: articulatory techniques, adequately selecting among them to achieve the best result according to the objective set Correctly carry out the following techniques of manual therapy: neurodynamic techniques, adequately selecting among them to achieve the best result according to the objective set Demonstrate management of pain – how is pain relieved? Demonstrate an understanding and implementation of pain management in a specific context/environment
		Referring to other providers Range of appropriate providers relevant to scope of practice and considerations for referral Typical eligibility criteria of providers relevant to scope of practice Potential costs and logistical requirements for accessing providers Referral pathways and procedures relevant to scope of practice, including information handover requirements	Referring to other providers Managing handovers Writing referrals
		Evaluating progress towards desired outcomes Expected trajectory of functioning with implementation of the rehabilitation plan relevant to scope of practice Range of outcome measures relevant to scope of practice and considerations for selection Intervals for evaluating progress towards desired outcomes Non-standardised approaches to determining progress towards desired outcomes, such as observation, self-report and family or caregiver perceptions Methods and techniques for using outcome measurement instruments relevant to scope of practice How to interpret and report outcome measures relevant to scope of practice	Evaluating progress towards desired outcomes Setting up and using equipment and consumables relevant to scope of practice Implementing inspecting, measuring and testing techniques Scoring standardised outcome measures Interpreting the results of outcomes measures Assessing body functions, activities and participation through observation and interview Evaluating outcomes Develop a working prognosis
		Discharging and ensuring appropriate continuity of care Information required and methods for determining discharge readiness, including typical indications and contraindications for discharge relevant to scope of practice Methods for determining the need for, and degree of, ongoing support and follow-up that a person and their family may require Approaches to facilitating self-management following discharge Potential logistical requirements for discharge or transition of care How to construct a discharge report, including key information points How to ensure successful transfer and/or storage of information on discharge	Discharging and ensuring appropriate continuity of care Managing handovers Closing relationships with a person and their family
	Communication	Prepare comprehensive and accurate health records and other documents, appropriate to purpose Adjust communication strategy consistent with purpose and setting Use appropriate terminology Adjust communication based on level of understanding of recipient Share information empathetically and respectfully Use electronic technologies appropriately and responsibly Effectively engages in interprofessional communication that positively affects patient outcomes within the speciality area of practice	Speak clearly and concisely Listen actively, build trust and foster exchange of information Use and respond to body language appropriately Give and receive feedback in a constructive manner Communicate timely Use images, videos and other media to enhance communication
	Clinical reasoning	Efficiently and strategically gathers, interprets and synthesises essential, accurate and disconfirming information from multiple resources in order to make more effective clinical judgements	Evaluates evidence-based practice, physical therapist expertise, and patient’s perspective and value in management of patient’s needs across varied practice settings or diverse patient populations Effectively reflects upon the application of evidence and modifies accordingly Presents a logical rationale for clinical decisions with patients, colleagues and the interprofessional team, while incorporating patient’s needs and values, within the context of ethical clinical practice Responds to anticipated and unanticipated outcomes in both simple and complex clinical conditions across varied practice settings or diverse patient populations
	Health promotion and prevention	Understand the role of patient education Understand the impact of social, cultural and behavioural variables on patient learning Integrates evidence-based practice into patient education Identify the determinants of health using a biopsychosocial and human right approach Use a biopsychosocial and human rights approach when applying health promotion and prevention strategies Contribute to planning and implementation of health promotion and prevention activities to improve population and individual health Understand community-based rehabilitation strategy	Provide education within limits of practice, seeking advice or referring to another professional where appropriate Empower patients to facilitate health behaviour change through motivational interviewing and implementation of self-management strategies Know, design and implement educational programmes for the health of the patient in different situations (chronic diseases, pain, risk groups, etc.) Consistently and regularly review progress of patient learning Utilise reflective questioning Use shared decision-making Promotes public health Be able to implement community-based rehabilitation in rural communities
Professional growth and involvement	Ethical, professional and interprofessional practice	Recognise the limitations of their own competence and ensure to work within it Recognise clinical and environmental risk Understand cultural humility, cultural competence, diversity, person-centred approach, privacy, autonomy and human rights Understand the laws and regulations governing the practice of physiotherapy as an autonomous profession, and the AHPC, ethical and professional codes, standards, guidelines and policies of NSP, AHPCN, HPCN Incident reporting policies and procedures How to conduct a risk assessment Recognise the need for, and implement, appropriate strategies to manage their physical and mental health and resilience Be an advocate for patients and the profession Be aware of one´s own role and the role of others within a multi-professional team Be aware of one’s own limitations on knowledge and skills Be aware of potential conflicts within a multi-professional/interdisciplinary team, and contribute to resolving problems Recognise autonomy and individuality of team members, while respecting diversity Definitions and principles of task-sharing and interprofessional practice Conscious and unconscious biases and personal beliefs	Obtain informed consent prior to intervention and respect the right of the client to refuse intervention Conducting risk assessments Using infection prevention and control measures, including donning and doffing personal protective equipment and performing hand hygiene Strategies to prevent and manage situations of conflict and violence, including de-escalation techniques Practise using a culturally competent, person-centred approach with respect for all forms of inclusion, diversity, dignity, privacy, autonomy and human rights of the client, or legal guardian, who is seeking services regardless of whether the services are provided in person or remotely Manage risk responsibly and effectively, and advocate for the right of physiotherapists to work in a safe and healthy practice environment that assures their own health and safety as well as that of their clients Engage actively in anti-corruption, global health and human rights-based approaches Practise within their own scope of practice; provide honest, competent and accountable professional services; refuse to work outside of their own competence, if requested to do so; and to accept responsibility for the exercise of sound professional judgement Report any observed unethical behaviours/practice by others – this includes digital practice, digital data protection and the use of social media Place the needs and interests of the client at the centre of their practice; provide fair, equitable, inclusive and empowering quality services and ensure their own needs and interests as a physiotherapist do not compromise practice; charge and receive a just and fair level of remuneration for their services Share relevant information with other team members while ensuring patient confidentiality Demonstrate effective team working for efficient case management and optimal service delivery
	Collaboration	Identify practice situations that may benefit from collaborative care Recognise and respect the roles of others Understand accepted principles for teamwork Recognise conflict or potential conflict, and respond constructively	Engage in an inclusive, collaborative, consultative, culturally responsive and client-centred model of practice Share information about the physiotherapist’s role and knowledge Negotiate shared and overlapping roles and responsibilities Interact with others in a manner that promotes inclusion Participate in team evaluation and improvement initiatives Apply conflict resolution principles in a structured fashion
	Quality improvement	Undertaking quality improvement initiatives Understand organisational data collection, interpretation and analysis to measure quantity and quality of outputs	Coordinating and evaluating quality improvement activities Engage with, and initiate, service improvement initiatives, including acting on feedback from clients Utilise resources and technology efficiently to ensure their maximal impact on services
Learning and development	Self-learn and develop	Recognise continuing education requirements for registration and licensing Understand principles and practices of self-directed learning Identify existing or potential opportunities for learning and development, and how to access them Understand different learning styles and how to identify and respond to them	Establishing and managing a professional development plan Appraising own professional performance Reflect on practice and seek support where needed to improve and develop one’s own personal and professional efficacy and effectiveness Identify learning needs related to the use of technology in physiotherapy including new diagnostic, intervention, communication and documentation tools addressing privacy, security, data storage, technology troubleshooting and adverse events management
	Supports the learning and development of others	Principles of adult learning Potential barriers to learning and development, and strategies to address these Responsibilities and obligations as a teacher or supervisor Range of resources, including existing and emerging technology, to support teaching and learning and how to use them Teaching and supervision techniques and modes of education	Construct and implement a personal development plan and engage in continuing professional development Identify individual learning needs by assessing one’s own practice against peers and benchmarks, and set realistic learning goals Designing training courses, including defining learning outcomes, modes of content delivery, assessment and evaluation Providing constructive feedback Using different modes of teaching Using different resources and technologies to enhance teaching Performance appraisal of others Contribute to clinical supervision of undergraduate students
Management and leadership	Managing a rehabilitation team	Understand: The scopes of practice, responsibilities and performance standards for health workers relevant to service context Different levels of monitoring and supervision, delegation, accountability and indications for applying these Strategies for team communication and coordination	Organise and prioritise their workload and resources to provide safe, effective and efficient physiotherapy autonomously and, where relevant, as a team member Allocating tasks Delegating responsibilities Rostering team members Scheduling appointments Identifying strengths and limitations of team members and how to manage these to best effect
	Managing rehabilitation service delivery	Understand: Epidemiological and demographic trends driving rehabilitation need relative to context Safe working conditions and related standards and regulations Principles of inclusive design and standards and regulations for accessibility Policies and legislation for human resource management Potential resource requirements for delivering services Strategies to mobilise resources and manage a service budget Methods of managing confidential information and related standards and regulations	Conducting stocktakes of assistive products, equipment and consumables Procuring resources Maintaining inventories Drafting policies and procedures
	Monitoring and evaluating rehabilitation service delivery	Have knowledge on: Service delivery indicators and associated data requirements and sources Potential service performance indicators and considerations for selection How to apply, interpret and report service performance measures Mechanisms for service data collection and aggregation Policies and regulations for data collection and reporting The structure and functions of the health information system and how rehabilitation is or could be integrated Methods of engaging rehabilitation service users in service evaluation Policies and procedures for conducting or coordinating service audits	Have skills on: Maintain confidentiality of records and data, with appropriate access Report writing Data collection, analysis and reporting, including data visualisation Using standardised service outcome measures Inputting into health information systems Conducting surveys Utilising resources effectively and in line with professional standards and code of ethics
	Contribute to leadership in the profession	Understand the role of the physiotherapist within the function and structure of the health system Understand quality improvement initiatives within the profession and/or the organisation	Provide for the ongoing growth and development of the profession and for the identification of the unique contribution of physiotherapy and its evolving scope of practice Advocate for accessibility and sustainability of physiotherapy and other services across the continuum of care Lead effectively and be led by others, as appropriate, and proactively model best professional values, and ethical behaviours Promote a culture of client-centredness
Research	Designing and implementing research	Understand critical synthesis of research literature to identify gaps Ethical standards for research with human subjects Potential sources of conflicts of interest and how these can be detected and managed Potential research grants relevant to context and how to access them Quantitative and qualitative study designs Types of research bias and how to mitigate for them Inferential and descriptive statistics Principles of ethical and respectful use of data, and relevant legislation and protocols	Use the best available evidence and new knowledge to inform and adapt practice to ensure it is safe and effective Constructing research proposals and protocols Writing ethics applications Collecting data from a range of sources Analysing quantitative and qualitative data, including use of statistical software Extracting meaningful conclusions from data and identifying potential applications Academic writing Use reliable and valid outcome measures to evaluate practice and modify accordingly
	Disseminating evidence	Impact factors and target audience of scientific journals Real or potential platforms for disseminating evidence Dissemination strategies for evidence	Writing scientific manuscripts Presenting evidence to different forums and in different formats
	Strengthening rehabilitation research capacity	Existing research capacity Barriers and facilitators to the expansion of research activities Rehabilitation research stakeholders and their respective roles The rationale for rehabilitation research, including health, economic, educational and social benefits	Developing stakeholder networks for research partnerships

*Source*: American Physical Therapy Association 2020; Chartered Society of Physiotherapy 2020; Díaz-Mohedo et al. 2021; Edgar et al. 2020; Forbes et al. 2018; Health Professions Council of South Africa 2022; Hoeger et al. 2014; Martin et al. 2021; National Physiotherapy Advisory Group (NPAG) 2017; Physiotherapy Board of Australia & Physiotherapy Board of New Zealand 2015; Physiotherapy Board of New Zealand 2018; Republic of Namibia 2002, 2018; University of Namibia 2022; Ursula et al. 2017; WCPT European Region 2018; World Confederation for Physical Therapy 2011; World Health Organization 2020; World Physiotherapy 2021
